# The Gordian Knot That Is Patient and Public Involvement in Interprofessional Education

**DOI:** 10.5334/pme.2261

**Published:** 2026-09-04

**Authors:** Matthijs H. Bosveld, Dante N. Mulder, Lucija Gosak, Isabelle Huys, Cathy C. Kline, Matilde R. P. F. Leal, Mateja Lorber, Elsa F. Mateus, Pedro Morgado, Catarina Raposo-Lima, Adrijana Svenšek, Charlotte Verbeke, Marloes A. van Bokhoven, Ricardo J. O. Ferreira

**Affiliations:** 1 Care and Public Health Research Institute (CAPHRI), Department of Family Medicine, Maastricht University, Maastricht, The Netherlands; 2 School of Health Professions Education (SHE), Faculty of Health, Medicine and Life Sciences, Maastricht University, Maastricht, The Netherlands; 3 Maastricht University, Maastricht, The Netherlands; 4 Patient as a Person Foundation, Eijsden, Limburg, The Netherlands; 5 University of Maribor, Faculty of Health Sciences, Maribor, Slovenia; 6 Department of Pharmaceutical and Pharmacological Sciences at KU Leuven, Leuven, Belgium; 7 University of British Columbia, Vancouver, Canada; 8 Health, Patient & Community Partnerships for Education, Vancouver, Canada; 9 Nursing Research, Innovation and Development Centre of Lisbon (CIDNUR), Nursing School of Lisbon (ESEL), Lisbon, Portugal; 10 National Institute of Public Health, Ljubljana, Slovenia; 11 Board of EUPATI Portugal, Lisbon, Portugal; 12 School of Medicine, University of Minho, Braga, Portugal; 13 Hospital de Braga, Braga, Portugal; 14 Instituto de Saúde Ambiental (ISAMB), Faculdade de Medicina, Universidade de Lisboa, Lisbon, Portugal

## Abstract

**Introduction:**

The combination of patient and public involvement (PPI) and interprofessional education (IPE) is promising to help students delineate roles based on real patients’ needs; however, their integration in practice remains limited. Although the individual concepts have been studied, little is known about how stakeholders perceive PPI in IPE, leaving gaps in designing effective educational strategies. Therefore, this study aims to explore the experiences and perspectives of key stakeholders – students, academic staff members, and patient representatives – on the feasibility, (intended) learning outcomes, and requirements for the successful implementation of PPI in IPE in undergraduate healthcare education.

**Methods:**

We qualitatively explored the experiences and perspectives of each stakeholder group in separate focus groups. In total, 15 focus groups were conducted across four European countries, involving 27 academic staff members, 27 students, and 24 patient representatives, all with diverse backgrounds. Data were analyzed using thematic analysis.

**Results:**

Themes were structured by organizational levels within educational institutions to optimize uptake in practice. As for strategic considerations (*align*), participants noted compounded organizational complexity, which may lead to inertia – especially without institutional support. At the same time, PPI in IPE should be valued equally compared to biomedical subjects. With respect to educational *design* considerations, PPI in IPE helped students bridge theory and practice and decreased hierarchy. Lastly, regarding operational considerations (*execute*), the compounding complexity of organizing patient involvement in interprofessional education also holds true for facilitating it, as facilitators must role-model both person-centered communication and interprofessional collaboration. According to participants, learning from PPI in IPE is about unifying what cannot be separated.

**Discussion:**

Despite strong rhetorical endorsement, a lack of institutional support persists. Our findings suggest PPI in IPE as a powerful means of addressing epistemic injustice by promoting epistemic symmetry between future professionals and patients, reducing interprofessional hierarchies, and grounding interprofessional learning in real-world experiences. Strategic use of these levers may unlock the necessary institutional resources, acting as Alexander’s sword to overcome barriers and ultimately slice through the Gordian knot.

## Introduction

The integration of patient^1^ and public involvement (PPI) in interprofessional education (IPE) remains under-implemented, underexplored, and underfunded despite fulfilling both core educational objectives and institutional mandates for social accountability. Although PPI and IPE have been well studied as standalone strategies, we do not yet understand how key stakeholders perceive the combination of PPI in IPE in terms of (intended) learning outcomes, feasibility, and requirements for successful implementation. In the absence of such understanding, important gaps persist in the design of effective educational strategies to bring PPI and IPE together. This matters as the meaningful integration of these concepts helps students develop the competencies necessary to deliver person-centered care, which might otherwise be difficult to achieve in undergraduate education [1,2].

IPE brings together students from different professions to learn with, from, and about each other, fostering teamwork and improving the quality of care [3,4]. As high-quality care is inherently patient-centered, it follows logically to actively involve patients and other members of the public in such education. PPI in undergraduate education concomitantly enables students to gain firsthand insight into patients’ experiences, thereby grounding IPE in authentic professional realities [5,6,7].

When PPI is meaningfully integrated with IPE, students’ learning becomes more realistic. They gain a deeper appreciation of how different professions work together to address complex, multifaceted patient needs through distinct yet complementary roles [8,9,10,11,12]. Patients bring authenticity through real-life experiences that foster meaningful connections between students and patients, leading to enhanced student empathy, communication, teamwork, and professional identity formation [8,13,14,15], while benefiting patients through generativity, illness acceptance, personal growth, and improved ability to express preferences [16,17].

Despite these clear theoretical and practical benefits, their integration in undergraduate healthcare education remains limited. Both IPE and PPI, when implemented separately, face well-documented barriers, including pedagogical challenges, logistical limitations, and threats of tokenism and epistemic injustice [13,18,19,20,21,22,23,24,25]. Combining the barriers and challenges of IPE with those of PPI might make PPI in IPE much like a Gordian Knot – a highly complex knot that seems impossible to untie.

Little research has systematically examined the (intended) learning outcomes, feasibility, and requirements for the successful implementation of PPI in IPE. To address this gap, this study aims to explore the experiences and perspectives of key stakeholders across Europe – students, academic staff, and patient representatives with diverse backgrounds – regarding feasibility, (intended) learning outcomes, and requirements for the successful implementation of PPI and IPE in undergraduate healthcare education.

## Methods

Situated within the interpretivism paradigm, we conducted qualitative research using focus groups to explore the experiences and perspectives of key stakeholders regarding PPI and IPE in undergraduate healthcare education. Focus groups were chosen to capture diverse experiences and perspectives on this topic, allowing in-depth exploration of these experiences and perspectives [26]. The study was reported following the Consolidated Criteria for Reporting Qualitative Research (COREQ) [27].

### Setting, participants, and sampling

Separate focus groups were conducted with three stakeholder groups: academic staff, students, and patient representatives. Academic staff included researchers, clinicians, teachers, and supporting staff from higher education institutions, such as universities, universities of applied sciences, and vocational educational institutions, with experience in PPI or IPE. Students included undergraduate students from the first to the fifth year. Both academic staff and students represented various disciplines, primarily nursing and medicine, as well as pharmacy, dentistry, and psychology. Patient representatives were members of national, regional, or local patient organizations. Representatives, rather than patients, were chosen because of their broader experience and ability to represent larger patient groups [28].

The focus groups were organized by the PULPIT research consortium partners: KU Leuven, Belgium (BE); Maastricht University, the Netherlands (NL); University of Minho and the Nursing School of Lisbon, Portugal (PT), and University of Maribor, Slovenia (SI). Each partner conducted one focus group per stakeholder group, totaling fifteen focus groups, in the native language. The focus groups were held partly on-site and partly online, using Zoom or Microsoft Teams, depending on the system supported by the educational institution.

The participants were recruited through purposive sampling to ensure diverse experiences and perspectives (e.g., varied educational backgrounds among students, experience with IPE or PPI among academic staff, and various health conditions among patient representatives). Academic staff were recruited through the researchers’ networks in the respective countries and contacted via email. Students primarily came from the institution where the researchers worked and were contacted via email and through announcements on online student platforms. Patient representatives were contacted through patient organizations, mostly via email or phone.

### Data collection

A topic list was created using the Krueger and Casey [29] guide and discussed with the research consortium. The topic guide was adapted for each stakeholder group, following a similar structure (Appendix I). A patient representative (EM) from the PULPIT consortium reviewed the topic list for patients, and alterations were made accordingly. The first focus group, conducted by MB and DM in the Netherlands in April 2024, with patient representatives, served as a pilot test for the topic list. This first focus group was still considered for analysis because of the richness of the data. Subsequently, the research team members carefully translated the topic list into the national language of the participating countries.

Moderators and facilitators from different institutions were prepared for the focus groups through two online meetings organized by Maastricht University, in accordance with AMEE guidelines, to optimize uniformity across countries [30]. One session focused on participant recruitment, while the second focused on moderating focus groups. A manual, aligned with the preparatory meetings, guided the moderators and facilitators in ensuring a uniform structure for the focus groups across different countries (Appendix II).

Written informed consent was obtained prior to the focus groups, either written for on-site focus groups or via email for online focus groups. In each country, two researchers conducted the focus groups. Representatives from the research consortium partners moderated the sessions, and when facilitators were present, they took field notes during the focus groups. In BE, the focus groups were moderated by KA and IH; in NL, by MB and DM; in PT, by RF and CL; and in SI, by LG and AS.

All focus groups were conducted between April and December 2024. The focus groups were recorded, and the recordings were transcribed verbatim and pseudonymized locally. Local researchers translated transcripts into English. After transcription, the audio recordings were deleted for privacy reasons. The pseudonymized transcripts were sent encrypted to the data-analysis team at Maastricht University, in accordance with the signed data-sharing agreements. Data were stored on the GDPR-compliant drive of Maastricht University, and data analysts were the only ones with access to all translated transcripts.

### Data analysis

Participants received a summary of the focus group in their native language within 14 days and were allowed to comment on or add to the summary as a member check. The coding of translated transcripts was performed independently by DM and MB, who also conducted the focus groups in the Netherlands and were therefore familiar with (parts of) the data.

The data analysis began with inductive coding of the translated transcripts, following the steps of thematic analysis [31,32]. Thematic analysis is a method for analyzing qualitative data that, in our case, entailed searching transcripts to identify, analyze, and report patterns, coding these patterns, and from there constructing themes [33]. DM and MB iteratively reviewed and compared their progress, regularly addressed coding discrepancies, and, in turn, identified emerging themes. They developed, data-driven codes and themes were organized using ATLAS.ti software (Version 25.0.1.32924). Subsequently, the analysts arranged themes following an organizational framework (align, design, and execute), moving towards a more abductive coding process. Discrepancies were resolved through discussion and, if necessary, discussed with a third researcher, MvB.

Data sufficiency was addressed during analysis among the analysts, who examined both the analytical process and the richness of the data, and they ultimately reached unanimous agreement on data sufficiency [34]. An overview of the coding tree is provided in Appendix III. The preliminary themes were presented to the other partners of the PULPIT consortium, who indicated that they resonated with their experiences in the focus groups.

### Reflexivity

This study was conducted as part of the PULPIT research consortium, which seeks to strengthen PPI in undergraduate IPE. This commitment to PPI in IPE might have shaped our interpretative stance during the research, particularly given our prior exposure to subjects such as epistemic injustice and issues of institutional support and hierarchy. From an interpersonal reflexivity perspective, as posed by Olmos-Vega, Stalmeijer [35], the power dynamics between researchers, students, and patient representatives during data collection warrant reflection. Across all focus groups, the moderators and facilitators did not assess participating students’ study progress; therefore, students could discuss the topic freely. The same holds true for physician-patient relationships between researchers and participating patient representatives. However, because most focus groups were held at unfamiliar sites and many patient representatives were participating for the first time, they might not have felt fully at ease, which could have affected their ability to contribute freely. As for contextual reflexivity, cultural norms may also have influenced data collection. For example, in the Netherlands, where PPI is more common and sought after in education and research, patient representatives might have been better able to articulate their experiences than in Portugal or Slovenia, where PPI is less common and hierarchical structures are more prevalent.

The data-analysis team consisted of three researchers. MB is a medical doctor and PhD candidate, with prior experience in qualitative research. Furthermore, he co-founded the Patient as a Person Foundation. During this study, he held no position there. DM is a medical student and a student assistant for the Patient as a Person Foundation. MvB is a professor in interprofessional collaboration and a general practitioner. Their complementary, sustained engagement with PPI in IPE enabled them to code the transcripts diligently and holistically, thereby deriving a complete and contextually grounded set of themes.

## Results

In total, 15 focus groups were conducted, involving 78 participants (27 academic staff members, 27 students, and 24 patient representatives). The focus groups lasted between one and a half and two hours. Following the focus groups, member checks were conducted, yielding a few additional remarks for the patient representative group in PT and the academic staff group in NL. In PT, this involved clarification about the patient organization’s activities, whereas in NL, a participant emphasized structural barriers to innovation and the implementation of education. Non-participation across all groups was primarily due to time and/or energy constraints.

The sample consisted of students from various healthcare education programs, predominantly nursing and medicine. Their age ranged from 18 to 24 years. The participating academic staff members had diverse backgrounds, with ages ranging from 25 to 65 years. The patient representatives covered a wide range of chronic diseases, including inflammatory bowel disease, cardiovascular disease, musculoskeletal and rheumatic diseases, lung disease, and various types of cancer. Two patient representatives held dual roles, serving as both patient representatives and students, with one pursuing a health-related study. Their age ranged from 30 to 75 years. A detailed overview of participants’ demographic characteristics is provided in Appendix IV.

**Figure 1:**
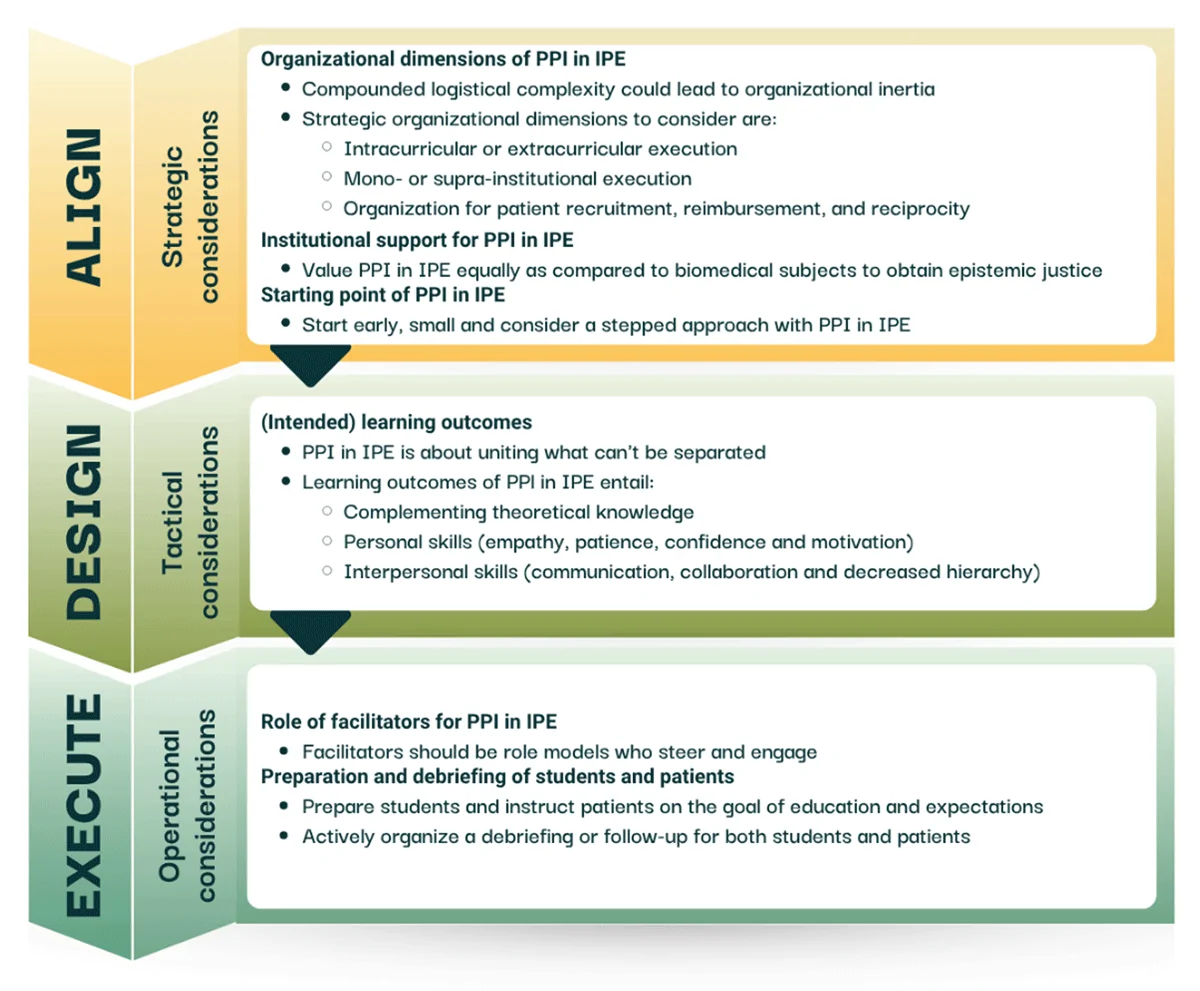
Overview of themes and recommendations following from them.

To facilitate readability and uptake in practice, the themes that emerged were structured into three levels for organizing education: ‘*align*’ – covering strategic considerations, ‘*design*’ – describing tactical considerations, and ‘*execute*’ – exploring operational considerations. We subsequently elaborate on these categories and how the themes are related. For each level, we will first focus on themes that are specific to PPI in IPE. Secondly, where applicable, we will elaborate on themes that surfaced but are not exclusive to the combination of PPI in IPE. An overview of the themes is presented in Figure 1.

### Themes

#### 1. Align

The align category concerns strategic considerations in education; topics that are typically discussed and decided on by senior leadership. Examples of these considerations include establishing long-term visions, goals, and policies to align institutions with societal needs and to ensure the sustainable development of education. Firstly, participants discussed the organizational dimensions of PPI in IPE and the associated logistical complexity. Secondly, participants discussed the need for institutional support and the starting point of PPI and IPE.

##### 1.1 Organizational dimensions of PPI in IPE

Participants discussed various dimensions of organizing PPI within IPE, highlighting the compounded logistical complexity of integrating PPI into IPE and the potential for subsequent organizational inertia. Notably, these different organizational dimensions led participants to question the feasibility of PPI in IPE ‘(*in the current environment and with the current demands placed on our students, it’s not feasible*, SI-A-2)’, despite acknowledging educational and societal advantages ‘(*the benefits are numerous*, SI-A-3)’. Participants discussed the dimension of (1) curricular or extracurricular execution, (2) mono- or supra-institutional organization, and (3) organization of patient recruitment, reimbursement, and reciprocity.

Participants weighed curricular implementation of PPI in IPE against offering it as an extracurricular activity and saw each constrained in different ways. As for implementation in curricula, PPI in IPE might not be considered a priority, as the existing curriculum already includes time-intensive subjects and students will eventually meet patients and other professions during internships. One participant mentioned: ‘*it’s harder to include patients at the faculty level, but it’s easier to do in a hospital environment’* (SI-S-7). When implementation in curricula seems infeasible, participants note that extracurricular execution remains an option. This was seen as a more feasible way to introduce new forms of education before implementation, since these initiatives involve smaller groups of students, thereby reducing coordination challenges. On the contrary, students have busy schedules and may not prioritize non-mandatory educational activities. Therefore, curricular implementation was stressed to have a broader reach, as you only reach *‘a specific group of students if you organize it [PPI in IPE] extracurricularly. Whereas I think it would actually be useful for all students.’* (NL-S-6). This highlighted the limitations of both routes to implementation: curricular integration was stressed as harder to achieve, while extracurricular activities reached only part of the student population.

In addition to deliberations within or outside the curriculum, participants considered at what level PPI in IPE should be organised, within a single institution (mono-institutional) or across institutions (supra-institutional). Students valued collaboration across faculties of the same institution: *‘collaboration between students from different faculties is very important*.’ (SI-S-6). Academic staff indicated that in education there are *‘institutional boundaries’* (NL-A-2), and referred to supra-organizational execution as *‘a very complicated issue in the current state of organization and cooperation between institutions’* (PT-A-3). The level of organization shaped how feasible collaboration was perceived, with supra-institutional cooperation perceived as more challenging.

An additional dimension of organization concerned the prerequisites for meaningful patient involvement: recruitment, reimbursement, and reciprocity. As for patient recruitment, diversity and representativeness were discussed by participants. Participants felt it was feasible to recruit and involve patients actively in IPE, but stated it is not *‘easy to draw a representative sample of all patients’* (BE-A-1) in education. Participants noted the potential to reduce organizational complexity of recruitment by collaborating with an external organization, such as a *‘study association’* (BE-S-1). Recruitment was thus seen as achievable but challenged by assembling a representative and diverse group.

Participants agreed on the value of compensating patients, but discussed reciprocity as opposed to financial reimbursement. The importance of compensation was supported by all participants, especially with *‘tokenism lurking’* (NL-A-2). Interestingly, financial compensation was not always deemed essential: reciprocity was considered the key concept. One patient found satisfaction in reading *‘what it [their participation in education] yielded, to see which eye-openers have occurred.’* (NL-P-2). An academic staff member echoed this, labeling financial remuneration as *‘a kind of quid pro quo. A transactional way of looking at it.’* (NL-A-2). Ideally, they posed, we could view PPI in IPE *‘relationally’*. Educators, students, and patients ‘*create quality in the moment together’*, each taking their own benefit from it. *‘The student could have a moment of growth, the patient could feel heard, or a thousand other things.’* (NL-A-2). This addressed the importance of compensation while exploring options beyond financial reimbursement.

##### 1.2 Institutional support for PPI in IPE

Participants discussed institutional support as a crucial prerequisite for implementing PPI and IPE in undergraduate HPE. They posed that *‘if [institutes] are not in on your story, (…) you can do whatever you like, but nothing or very little will change’* (BE-A-5). This was thought to be especially true in light of the compounding complexity of PPI in IPE: decision-makers must be convinced of the importance of integrating PPI in IPE. Participants stressed that *‘this type of education [PPI in IPE]’* should be *‘equally valued’*, as it occurs that the organization of PPI in IPE happens *‘in the spare time of the academic staff, whilst physiology and anatomy are done in regular hours’* (NL-A-5). Equally valuing these topics should be considered not only in terms of labor, but also conceptually. One participant addressed the topic of epistemic injustice, stating that too often *‘patients’ stories in a personal context are only seen as something you should treat decently at the most, or listen to from time to time’* (NL-A-2). This might indicate that PPI is seen as *‘an inferior source of knowledge compared to medical evidence-based knowledge’* and that academic communities *‘should dare to have a deeper conversation about what actually is quality of care and what forms of knowledge matter.’* (NL-A-2). Institutional support was seen to depend on how PPI in IPE was valued, both in the allocated time and the recognition of patient experiences as a legitimate form of knowledge.

##### 1.3 Starting point for PPI in IPE

Participants discussed when to start PPI in IPE, with each starting point demonstrating different benefits. An early start was thought to foster an interprofessional mindset and person-centred competencies from the start of their studies. Patients indicated that *‘the earlier it happens, the more impact it can have on the part of training and awareness’* (PT-P-2). Students echoed this and valued early exposure for connecting classroom learning to clinical reality, noting ‘*every patient is different, and you have to adapt to that. It would be great if we could understand this right from the start.’* (SI-S-7). However, a later introduction of both concepts was seen as more feasible, as students have begun to form a professional identity and have developed the knowledge and skills necessary to engage in patient contact. One student mentioned that ‘*in the first year we don’t have so many experiences to talk [about] to our colleagues’* (PT-S-6), supported by another student stating that students do not *‘have enough knowledge to work directly with patients before the fourth year’* (SI-S-4). Participants thus remained divided, with each timing providing different advantages.

Whether to start early or late with PPI in IPE was found to depend on the institution’s intended learning objectives. One participant mentioned that it *‘all depends on the goal you want to achieve’*, and that it could be beneficial for students early in education to ‘*just to hear a patient’s story of what they have been through to have a sense of what that might mean on an individual human life’* (BE-P-5). Another participant suggested introducing this form of education in stages and deepening the shared experiences as students gain more experience. A *‘stepped-approach’*, where one shares ‘*the least vulnerable piece [of their experience] with the least experienced students,’* and *‘you continue from that.’* (BE-A-5). The timing of PPI could thus be seen as a gradual process rather than a single moment, matched to the goals of the educational institution.

#### 2. Design

The design category encompasses tactical considerations that translate strategic objectives into actionable education, including program design, necessary for effective execution. We will elaborate on the (intended) learning outcomes for PPI in IPE.

##### 2.1 (Intended) learning outcomes of PPI in IPE

According to participants, involving patients in IPE was considered logical, since they are increasingly being approached as equal members of the healthcare team and it provides the team with a shared vision of the same goal. PPI in IPE was described as *‘uniting, bringing together, what can’t be separated’* (PT-P-5). The importance of PPI in IPE was addressed, because after all, *‘professionals should not only be talking about patients [amongst themselves]’* but *‘patients should have an equally active role in interprofessional education’* (BE-P-3). Participants noted, however, that *‘interprofessional education doesn’t always have to be with a patient’*, while a perceived advantage of involving patients helps students realise that ‘*we’re all in training to help this person, to improve their health.’* (NL-A-5), positioning patients as a unifying lens in IPE. This showed that participants did not see patient involvement in IPE as an optional element, but more as a reminder of the purpose of education.

All participants agreed that patient involvement in interprofessional educational programs enriched students’ learning by complementing theoretical knowledge and bringing abstract concepts to life based on realistic stories. This was said to be true for both the comprehension of *‘certain diseases or certain situations better’* (SI-A-6) as well as understanding *‘the relationship, the empathy, the understanding of the person behind’* and comprehending ‘*what it is to be a person with a disability’* (PT-A-9). According to patient representatives, these insights were not limited to the person with the disease, but also gave insight into the impact on the patient’s relative: *‘whole network of connections that this person has, especially family connections, and that [disease] has an impact [on them as well]’* (PT-P-6). Ultimately, PPI in IPE was believed to contribute to a better understanding of the impact of illness on patients and their relatives.

Beyond knowledge, PPI in IPE was said to contribute to students’ personal skills. It was associated with improved empathy and patience, as well as with enhanced motivation and improved self-confidence. One student described feeling *‘more confident’* (SI-S-2), and another felt it helped to develop more *‘develop more patience’* (SI-S-4). These qualities were seen as better fostered through this form of education than through theory alone.

PPI in IPE was also seen to strengthen students’ interpersonal skills. Academic staff members experienced enhanced person-centered communication in their students. One staff member mentioned that ‘*they [students] suddenly understand much better why we want that [type of communication] and what we mean by it’* (PT-A-6). Not only was PPI in IPE believed to contribute to patient-related communication, but it also improved interprofessional communication and collaboration with future colleagues. A student mentioned it lowered the threshold to reach out to one another in practice: *‘calls would be made faster’* (BE-S-2). This was also addressed by academic staff members: *‘it improves communication, builds trust among colleagues, improves cooperation, and I believe it could also raise the quality of patient care.’* (SI-A-1). Part of this was attributed to a decrease in perceived hierarchy. One academic staff member described a change in the students’ attitude:

‘*When they [students from vocational education] [are] with students from the university, or students of applied sciences, they feel very small. (…) When they complete the module, they feel enormously welcome. (…) They experience that they can really make a very valuable, um, valuable contribution. And that, that strengthens them enormously.’* (NL-A-1)

Altogether, PPI in IPE was considered to improve interpersonal skills, partly by improving skills needed for person-centred communication and partly by reducing perceived hierarchy.

#### 3. Execute

The execute category encompasses operational considerations that manage the day-to-day delivery of education. As for PPI in IPE, participants discussed the role of facilitators. Secondly, the need to prepare students and patients and to debrief was discussed.

##### 3.1 Role of facilitators for PPI in IPE

Participants noted that there is also compounded complexity in facilitating PPI in IPE because facilitators must combine several roles simultaneously. One of these roles included being a role model in person-centered communication, and one staff member noted that not every facilitator has ‘*the competencies on how to interact with patients*’ (BE-A-2). Furthermore, facilitators ought to *‘steer the conversation’* (NL-P-7) and be able to engage students at different levels within the module, fostering an environment in which students and patients can exchange opinions and experiences. A possible consideration in execution postulated by a student is *‘to have a tutor for each group of students*’, meaning that, ‘*in a group (…) of medicine and nursing [students], there is a doctor and a nurse in charge’* (PT-S-4). Facilitation was thus perceived as complex and as requiring the right competencies.

##### 3.2 Preparation and debriefing of students and patients

A universal concern among participants was students’ behavior towards patients, and subsequently they addressed the need for preparation. One participant noted that students must be reminded to maintain ‘*a posture and extremely correct behavior because we are facing a patient’* (PT-S-3). Preparation was also seen as a way to set expectations about what would happen and regarding ‘*privacy, confidentiality, and respect for the patient’* (PT-A-4).

As for the preparation of patients, academic staff indicated the importance of preparation, partly to align their story with the intended learning outcomes. One staff member noted: *‘what they [patients] want to tell, is not necessarily the same as what we [academic staff] want them to tell’* (PT-A-3). Preparing patients could inform them about the goal of the education, about the fact that they could decline to answer questions, and acquaint them with the level of the students. Patients are not always aware of these points: *‘We do not know what is already covered in the curriculum’* (NL-P-7). Furthermore, preparing patients gave institutions and patients a chance to check in on whether they are ready to participate in education: *‘for the patient, it’s trying to realize if it’s something they really want and are prepared for, because you end up exposing yourself a lot’* (PT-S-10). Preparing patients was thus seen to protect both the quality of the education and the patient.

Finally, participants suggested an active and plenary debriefing. This was meant to prevent students from *‘leaving with the wrong ideas’* (NL-A-3) and to provide support and *‘the necessary care’* for students, as *‘it’s not movies you show in your lessons, they are real people’* (BE-A-5). Concurrently, follow-up of patients should be considered to ask *‘how they experienced their involvement’* (BE-A-5) and to ensure we are not *‘over-exposing them’* (PT-A-4). For students as well as patients, debriefing was perceived as an important addition to provide support and care.

## Discussion

This study explored the experiences and perspectives of students, academic staff, and patient representatives regarding the (intended) outcomes, feasibility, and requirements for successful implementation of PPI in IPE within undergraduate HPE across four European countries. Our main findings highlight the need to manage diverse and compounding organizational deliberations – such as curricular implementation or extracurricular execution, mono- or supra-institutional organization, and the organization of patient recruitment and reciprocity in PPI in IPE. At the same time, patients ground interprofessional learning in reality as they learn personal and interpersonal competencies, whilst facilitators ought to be role models in both person-centered communication and interprofessional collaboration.

While the benefits and importance of PPI in IPE were widely recognized across all participating countries and stakeholder groups, its successful implementation was perceived as challenging, and a lack of institutional support persists. In their umbrella review, Gross and Ruelle conclude that PPI in (interprofessional) education suffers from a form of pedagogical liminality, stating that PPI being under-funded and under-implemented *‘cannot be rationally explained, given the well-documented benefits of this approach’* [36]. Similarly, Khalife and LaDonna point out that the structural incorporation of PPI into educational practice remains random and incidental, let alone in IPE. Therefore, they call for a shift from a rhetorical appreciation of patient involvement to the genuine integration of patients into our educational practices [37].

### PPI in IPE and epistemic injustice

Moving from rhetorical appreciation to genuine integration is only possible when key stakeholders in education are aware of different forms of *knowing* and position them on an equal footing. This discovery builds on the findings of our study, as participants noted that PPI in IPE might not be conceptually valued equally to biomedical subjects. When legitimate knowers are not believed because of their social identity, epistemic injustice occurs. This injustice can affect historical minorities, but it can also arise on the basis of gender, sexuality, race, age, or – within a healthcare context – the lived experiences of patients. Fricker [38] describes two main forms of epistemic injustice: testimonial injustice and hermeneutical injustice.

Testimonial injustice occurs at the individual level, between speaker and listener, when the listener’s prejudices lead them to dismiss or undervalue the speaker’s knowledge. Hermeneutical injustice arises when structural prejudice is so deeply embedded in the fabric of society that no shared interpretive resource exists to recognize or articulate the experience [39].

In healthcare, illness can lead to testimonial injustice when patients’ statements are ignored or dismissed, as health professionals often place greater weight on biomedical knowledge. It can also lead to hermeneutical injustice when patients lack the tools to adequately articulate their experiences of illness [40]. Yet patients possess unique and valuable perspectives and knowledge about what it is like to be ill and to receive care – an epistemic privilege for those who have not yet experienced this and are willing to listen, recognize, and value this knowledge.

Most of the research on epistemic injustice to date focuses on injustice on the patients’ end. At the same time, Bueter and Jukola argue that interprofessional teams are often characterized by medical dominance, which, in turn, can lead to so-called *institutional* epistemic injustice affecting both health professionals and, subsequently, patients [41]. On the professionals’ end, medical dominance may inflate medical doctors’ testimonies while deflating other professionals’ testimonies outside the medical domain, creating asymmetric credibility norms within the team. Subsequently, this can lead to epistemic injustice towards patients through *institutional opacity* [42]. Institutional opacity refers to a situation, e.g., a healthcare system, that is not transparent to its users, making it difficult to navigate and difficult to credibly express oneself. Patients might be unable to determine which professional to address for a given issue and may have to adapt their own expressions to align with the norms of different, unaligned professions.

To overcome challenges related to interprofessional collaboration, Bueter and Jukola suggest implementing interprofessional education to attenuate medical dominance and thereby advance epistemic justice. By combining their recommendation with the notion of the epistemic privilege that patients possess, PPI in IPE can promote (institutional) epistemic justice by strengthening epistemic symmetry between (future) professionals and patients.

### Alexander’s sword: pushing the right buttons to unlock institutional support

Although the rhetorical appreciation for PPI in IPE is widely recognized across countries and stakeholders, the compounded logistical complexity of integrating PPI into IPE still calls for much-needed institutional support. As our research shows, PPI in IPE is not a minor pedagogical tweak, but a multi-layered problem requiring a bold, paradigm-shifting institutional intervention. Our findings identified several levers that could unlock and sustain institutional support for PPI in IPE. Building on the previous paragraphs, a lever for institutional buy-in might be PPI in IPE to create a more level epistemological playing field.

Reducing hierarchy between professions through PPI in IPE could be another such lever. Participants in our study reported changes in their students’ attitudes toward other professions, indicating a decrease in perceived hierarchy. In a study of professional identity formation in an interprofessional health mentors program, professional hierarchies were attenuated during learning sessions with a patient mentor, as students discovered that, while they may bring different approaches to patient care, each has the patient’s best interests at heart [14].

Similarly, participants noted the opportunities for PPI in IPE to bridge theory and practice, helping students develop their personal and interpersonal skills. Following Fink’s interactive nature of significant learning [43], learning foundational knowledge and learning how to apply this can be enhanced by adding the human dimension. Therefore, learning from patients helps bring practical meaning to abstract concepts such as patient-centered care [6,7]. Enhanced interpersonal skills and personal growth have been well-documented outcomes of PPI for students [13,44,45].

Enhancing interpersonal skills, reducing hierarchy between professions, and creating a more epistemologically level playing field can generate institutional buy-in and serve as Alexander’s sword to slice through the Gordian Knot, thereby unlocking the resources needed for leadership, recruitment, coordination, facilitation, and assessment of PPI in IPE.

### Limitations

Although we conducted thorough research, this study has limitations. The level of experience with PPI in IPE varied across participating countries, so not all participants drew on their own experience, leading to more general remarks. Therefore, we mention both experiences and perspectives. In addition, the authors acknowledge the potential for selection bias resulting from the purposive sampling method adopted. This approach could have led to participants with a more positive attitude towards PPI and/or IPE, thereby influencing the findings. Moreover, the students’ sample consisted mainly of students with backgrounds in medicine and nursing, possibly limiting its transferability to other professions. Although most focus groups were conducted in person, some focus groups were held online. This approach may have negatively affected group dynamics and, in turn, the richness of the data [46]. Furthermore, the transcripts were translated by research team members rather than professional translators. Although they have professional backgrounds in the subjects discussed, this fact could have affected the linguistic and conceptual accuracy of the transcripts and, in turn, influenced the interpretation of the data. Lastly, due to the international, decentralized execution across four countries and three languages, an iterative approach between data collection and analysis was not adopted, potentially hindering the authors’ ability to gain a deeper understanding within each focus group.

## Conclusion

The study explored the experiences and perspectives of key stakeholders – students, academic staff, and patient representatives – on patient and public involvement in interprofessional education in health professionals’ education across four European countries. Compounded logistical complexity, patients serving as a unifying lens in interprofessional education, and facilitators having to role-model both person-centered communication and interprofessional collaboration are important considerations for implementing patient and public involvement in interprofessional education. Pulling the right *levers*, such as creating a more epistemologically level playing field, reducing hierarchy, and creating significant learning experiences, might help unlock institutional support, therefore acting as Alexander’s sword and helping to slice through the Gordian Knot. Ultimately, longitudinal studies on the impact of PPI in IPE will be needed to help sustain institutional support.

## Additional File

The additional file for this article can be found as follows:

10.5334/pme.2261.s1 Supplementary Material. Appendices I to IV.

## Data Availability

The data supporting this study’s findings are available from the corresponding author upon reasonable request.

## Footnotes

Throughout this paper, the term ‘patient’ is used. We aim to be inclusive of service-users, consumers, experts by experience, carers and/or family members, although the important distinctions between these terms are acknowledged. We recognize that no single term is adequate or universally acceptable.
