# Supplementary material for: The Gordian Knot That Is Patient and Public Involvement in Interprofessional Education
Source: Perspect Med Educ. 2026 Sep 4;15(1):705–17. doi: 10.5334/pme.2261 (PMC13544041; doi:10.5334/pme.2261)
Supplement: Supplementary file 1 [file pme-15-1-2261-s1.pdf]

Appendix I - Topic lists

Academic staff

Opening question (short, concise answers; 30s per participant!)

1. In what way are you involved in either interprofessional education or actively involving patients in health professions education?

Introductory question (allow participants to talk about how they see or understand the issue)

1. What are some important considerations with respect to your work in either interprofessional education or actively involving patients in health professions education?

Transition questions (setting the stage for productive key questions)

Attitudes towards interprofessional education

1. Is interprofessional education important within health professions education? If so, why? If not, why not?
2. What are critical factors in successfully teaching interprofessional competencies to optimally obtain intended learning outcomes?
    i. Do you think it is beneficial to include students from different professions *within* the university/university of applied sciences? If so, why?
    ii. Do you think it is beneficial to include students from different professions from different levels of education? If so, why?

Attitudes towards actively involving patients in education

1. Is actively involving patients in health professionals education, for example in regards to teaching person-centred care competencies, important within health professions education? If so, why? If not, why not?
2. What are critical factors in successfully involving patients in health professionals education?
    i. Do you think it is beneficial to actively involve patients to this end? If so, why? If not, why not?
    ii. To what extent can patients actively be involved in education? Why (not)?

Key questions (these are the questions that drive the study, reserve time for this part!)

Active involvement of patients in interprofessional education

1. Would actively involving patients in interprofessional education be feasible? If so, why? If not, why not?

2. From your experience, if present, what does combining active involvement of patients in interprofessional education result in for students/patients?
    a. If no experience is present, what do you expect it might be the result for students/patients?
    b. What are the necessary prerequisites to combine these goals?
        i. In terms of interprofessional education
            1. Assessment
            2. Formation of professional identity
            3. Practical implications and organisation
        ii. In terms of active involvement of patients
            1. Traits and training
            2. Practical implications and organisation
            3. Reciprocity and the role of educational institutions

Closing question (enable participants to reflect back on previous comments)

1. In an ideal world: would you implement interprofessional education actively involving patients? If so, why? If not, why not?
2. Is there anything that you would like to say that we did not cover?

Patient representatives

Opening question (short, concise answers; 30s per participant!)

1. Is your organisation already involved in educational initiatives for health professions education?

Introductory question (allow participants to talk about how they see or understand the issue)

1. In what way are you involved in health professions education? Are there initiatives that are either interprofessional in nature or initiatives where patients are actively involved?
2. What are your goals in participating in education?

Transition questions (setting the stage for productive key questions)

Attitudes towards person-centred care

1. What is person-centred care according to you? Is it important? If so, why? If not, why not?
2. What are your experiences in regards to person-centred care in practice? What is the current 'gap' that could be closed in education?
3. In your eyes, what should educational institutions focus on when education person-centred competencies?
    a. Do you think it is beneficial to actively involve patients to this end? If so, why? If not, why not?
    b. What do you think patients actively involved in education have to offer to undergraduate students in health professions education?

Attitudes towards and experience with interprofessional collaboration

1. Is interprofessional education important? If so, why? If not, why not?
2. What are your experiences in regards to interprofessional collaboration in practice? What is the current 'gap' that could be closed in education?
3. In your eyes, what makes interprofessional education a success?
    a. Do you think it is beneficial to include students from different professions *within* one institution, or from *different* institutions? If so, why? If not, why not?

Key questions (these are the questions that drive the study, reserve time for this part!)

Active involvement of patients in interprofessional education

1. Would combining interprofessional education and active involvement of patients be feasible in education? If so, why? If not, why not?
2. What are the necessary prerequisites to combine these goals for patients?

a. In terms of interprofessional education
    i. Tailoring your lessons to different professions
    ii. Organisation
b. In terms of active involvement of patients
    i. To what extent do you think patients would like to be involved?
    ii. What traits are necessary in patients to have them achieve intended learning outcomes?
    iii. In what way can educational operationalise reciprocity to participating patients?

Closing question (enable participants to reflect back on previous comments)

1. In an ideal world where you are in charge of education: would you implement interprofessional education actively involving patients? If so, why? If not, why not?
2. Is there anything that you would like to say that we did not cover?

Students

Opening question (short, concise answers; 30s per participant!)

1. Do you get in contact with students from other professions or do you get in contact with patients in your education?

Introductory question (allow participants to talk about how they see or understand the issue)

1. In what way are you involved in interprofessional education or are patients actively involved?

Transition questions (setting the stage for productive key questions)

Attitudes towards and experience with interprofessional education

1. Do you think interprofessional education is important within your education? If so, why? If not, why not?
2. What do you learn at your institution in regards to interprofessional education? What would you want to learn (additionally)?
3. In your eyes, what makes interprofessional education a success?
    a. Do you think it is beneficial to include students from different professions *within* your institution? If so, why? If not, why not?
    b. Do you think it is beneficial to include students from different professions from *other* institutions? If so, why? If not, why not?

Attitudes towards person-centred care

1. Do you think it is important to actively involve patients, for example to foster person-centred care competencies, in your education? If so, why? If not, why not?
2. What do you learn at your institution in regards to person-centred care? What would you want to learn (additionally)?
3. In your eyes, what makes teaching person-centred care a success?
    a. Do you think it is beneficial to actively involve patients to this end? If so, why? If not, why not?
    b. What would you want to learn from patients actively involved in education?

Key questions (these are the questions that drive the study, reserve time for this part!)

Active involvement of patients in interprofessional education

1. Would combining interprofessional education and active involvement of patients be feasible? If so, why? If not, why not?
2. From your experience, if present, what does combining active involvement of patients in interprofessional education result in for students/patients?

   a. If no experience is present, what do you expect it might be the result for you as a student/for participating patients?

3. What are the necessary prerequisites to combine these goals for you as a student?
   a. In terms of interprofessional education
      i. Assessment
      ii. Practical implications for students
   b. In terms of active involvement of patients
      i. Traits and training
      ii. Preparation of students
      iii. Psychological safety

Closing question (enable participants to reflect back on previous comments)

1. In an ideal world where you are in charge of your education: would you implement interprofessional education actively involving patients? If so, why? If not, why not?
2. Is there anything that you would like to say that we did not cover?

Appendix II – Manual for focus groups

What to do before, during and after the focus group

Before

1. **Welcome everyone and make a brief round of introductions**
2. **Introduce the topic**
    a. Today we are going to talk about your experience with and perspectives on actively involving patients in interprofessional education. Interprofessional education is a form of education in which two or more professions learn with, from, and about each other to improve the quality of care. Additionally, we will discuss person-centered care. This is care that is tailored to a person's personal needs, desires, and preferences. The goal of person-centered care is for individuals to live meaningful lives. We will discuss this later using a topic list.
3. **Explain the "ground rules" of a focus group**
    a. The information discussed within this session is strictly confidential and will remain within these four walls;
    b. The conversation will be recorded so that it can then be fully transcribed for analysis;
    c. You may indicate at any time during the focus group that you wish to stop participating in the study. The recording will be used up to that point, including what you have said up to that point.
    d. Because the conversation will be recorded, I would ask you to let each other finish speaking, otherwise this will cause problems in processing/understanding the recording;
    e. Nodding 'yes' or 'no' will also not be heard. If you disagree or agree with something, try to speak it out;
    f. You will not be judged by what you say, we are interested in all experiences on the subject, both positive and negative;
    g. The goal is not to agree with each other (although it is allowed). We respect everyone's point of view;
    h. The facilitator is there to ask more profound questions and guide the conversation;
    i. Tell participants that we will have one break and that they can indicate if they need the break.
4. **Ask if there are any questions in response to the participant information, and answer any remaining questions that may remain**

5. **Explain the consent/informed consent form and have it signed in duplicate. Take one copy.**
6. **Have the demographic data form completed and assign codes.**
    a. It is essential to be able to match codes to quotes later in transcription and analysis
7. **Start the recording and briefly repeat what you performed in items 1 through 5.**

During

1. **Start the focus group by following the topic list**
    a. Keep the following in mind: Moderating a focus group is more than just reading the questions out loud.

       It is NOT the goal to be complete in the transition questions; **it is your goal to be complete in the key questions.**

After

1. **Write a member check and send it out within <u>14 days</u> after the focus group!**
    a. Additions or comments on the member check are part of research data and should be sent to corresponding using the provided link (see step 4).
2. **Transcribe the audio recording verbatim**
    b. Take the assigned code to each participant and link it to their voice. This way, relevant demographics can be interpreted by the audience when reading illustrative quotes in the results section.
3. **Translate the transcriptions into English**
4. **Sign the data-sharing agreement and ask the corresponding author for a link to send the data securely. <u>DO NOT SEND DATA PER E-MAIL.</u>**

    Send the following documents through the encrypted link provided:
    - Table including codes linked to demographics
    - Transcription in English in a Word-file
    - If applicable: an overview of comments or additions to the member check

5. **Safely store the informed consent forms following local regulations at your own institution**

Appendix III - Code tree

Code	Code Groups
● (Dis)advantage PPI for patients	(Perceived) (dis)advantages for patients
● Assessment students	Organization of education(al activities)
● Disadvantage extracurricular	Organization of education(al activities)
○ Example of education (other)	
● Example of IPE	Educational examples
● Example of PPI	Educational examples
● Example of PPI + IPE	Educational examples
● Example of SP	Educational examples
● Expected learning outcomes IPE	(Expected) Learning outcomes
● Expected learning outcomes IPE + PPI	(Expected) Learning outcomes
● Expected learning outcomes PPI	(Expected) Learning outcomes
● Expected learning outcomes SP	
● Experience outside education	Experience with education
● Experience with IPE	Experience with education
● Experience with PPI	Experience with education
● Experience with SP	Experience with education
● Facilitators & Barriers	Organization of education(al activities)
○ Gaps in current education	
○ Healthcare practice	
● Implications for practice IPE	Implication for practice
● Implications for practice IPE + PPI	Implication for practice
● Learning outcomes PPI	(Expected) Learning outcomes
● Organization of education(al activity)	Organization of education(al activities)
● Patient recruitment	Organization of education(al activities)
● Patient requirements	Organization of education(al activities)
● Perceived (dis)advantage PPI for patient	(Perceived) (dis)advantage for patients
○ Perceived (dis)advantage SP	
● Suggestions for education	

Appendix IV – Demographics of participants

Patient representatives

N	ID	Country	Gender	Age	Level of education	Marital status	Disease represented	Active treatment	Daily activities
**1**	BE-P-1	BE	Male	65-69	ISCED-7	Married or living together	(Non-life-threatening) cancer	Yes	Retired
**2**	BE-P-2	BE	Male	65-69	ISCED-6	Married or living together	(Non-life-threatening) cancer	Yes	Paid work
**3**	BE-P-3	BE	Female	25-29	ISCED-7	Married or living together	N/A	N/A	Paid work
**4**	BE-P-4	BE	Male	70-74	ISCED-2	Single	Lung disease	Yes	Retired
**5**	BE-P-5	BE	Male	65-69	ISCED-7	Married or living together	(Non-life-threatening) cancer	No	Retired, volunteering
**6**	NL-P-1	NL	Male	65-69	ISCED-6	Married or living together	Lung disease	Yes	Retired
**7**	NL-P-2	NL	Female	45-49	ISCED-6	Married or living together	Inflammatory bowel disease	Yes	Paid work
**8**	NL-P-3	NL	Female	45-49	ISCED-6	Married or living together	Lung disease	Yes	Unfit to work, volunteering
**9**	NL-P-4	NL	Female	35-39	ISCED-6	Single	Neuromuscular disease	No	Self-employed
**10**	NL-P-5	NL	Female	55-59	ISCED-6	Married or living together	Cerebrovascular disease	Yes	Unfit to work, volunteering

**11**	NL-P-6	NL	Female	55-59	ISCED-6	Married or living together	Neurological disease	No	Unfit to work
**12**	NL-P-7	NL	Female	45-49	ISCED-7	Married or living together	Lung disease	Yes	Unfit to work
**13**	NL-P-8	NL	Female	75-79	ISCED-6	Married or living together	(Non-life-threatening) cancer	No	Retired, volunteering
**14**	PT-P-1	PT	Female	40-44	ISCED-6	Single	Neuromuscular disease	Yes	Unemployed, volunteering work
**15**	PT-P-2	PT	Female	45-49	ISCED-6	Does not want to disclose	Musculoskeletal disease	Yes	Unemployed, volunteering work
**16**	PT-P-3	PT	Female	45-49	ISCED-6	Single	Musculoskeletal disease	Yes	Student, volunteering
**17**	PT-P-4	PT	Female	35-39	ISCED-7	Married or living together	Cerebrovascular disease	No	Paid work
**18**	PT-P-5	PT	Female	55-59	ISCED-6	Married or living together	(Non-life-threatening) cancer	No	Paid work
**19**	PT-P-6	PT	Female	50-54	ISCED-6	Divorced	Inflammatory bowel disease	Yes	Paid work
**20**	PT-P-7	PT	Female	45-49	ISCED-6	Divorced	Musculoskeletal disease	Yes	Paid work
**21**	SI-P-1	SI	Male	30-34	ISCED-7	Married or living together	Prevention	No	Paid work
**22**	SI-P-2	SI	Male	50-54	ISCED-6	Married or living together	(Non-life-threatening) cancer	No	Paid work
**23**	SI-P-3	SI	Female	70-74	ISCED-3	Married or	Musculoskeletal disease	No	Retired

						living together			
**24**	SI-P-4	SI	Male	30-34	ISCED-6	Single	Prevention	No	Student

Academic staff

N	**ID**	**Country**	**Gender**	**Age**	**Level of education**	**Field**	**Role(s)**	**Area of expertise**
**1**	BE-A-1	BE	Female	55-59	ISCED-8	Sociology	Educator and researcher	Sociology of health and wellbeing
**2**	BE-A-2	BE	Male	50-54	ISCED-8	Medicine	Educator and researcher	Nephrology
**3**	BE-A-3	BE	Male	55-59	ISCED-8	Bioscience engineering	Educator and researcher	Human health engineering
**4**	BE-A-4	BE	Female	55-59	ISCED-8	Educational sciences	Educator and researcher	Educational sciences, health literacy education
**5**	BE-A-5	BE	Female	30-34	ISCED-8	Social Sciences	Researcher	Patient participation in care settings
**6**	NL-A-1	NL	Female	60-64	ISCED-6	Nursing	Educator	General nursing
**7**	NL-A-2	NL	Male	45-49	ISCED-7	Medicine	Educator and researcher	Transformative learning
**8**	NL-A-3	NL	Female	45-49	ISCED-7	Medicine	Educator	Clinical skills and communication
**9**	NL-A-4	NL	Female	60-64	ISCED-3	Service & support	Support staff	Contact with simulated patients in communication and reflective education
**10**	NL-A-5	NL	Female	55-59	ISCED-7	Medicine / Pharmaceutics	Educator and researcher	Patient participation, faculty development
**11**	NL-A-6	NL	Female	35-39	ISCED-7	Educational sciences	Educator and researcher	Pedagogic, boundary crossing in education
**12**	PT-A-1	PT	Female	45-49	ISCED-8	Nursing	Educator and researcher	Palliative care, medical-surgical nursing, educational sciences

**13**	PT-A-2	PT	Female	50-54	ISCED-7	Pharmacy	Educator and researcher	Clinical pharmacy, pharmacotherapy, health, and environment
**14**	PT-A-3	PT	Male	50-54	ISCED-8	Nursing	Educator	Rehabilitation, clinical supervision
**15**	PT-A-4	PT	Male	45-49	ISCED-8	Medicine	Educator and researcher	Assessment, simulation, intensive care medicine, and pain
**16**	PT-A-5	PT	Female	60-64	ISCED-7	Nursing	Educator	Chronic illness, older adults, and clinical simulation
**17**	PT-A-6	PT	Female	40-44	ISCED-7	Medicine	Educator and physician	Clinical communication, general practice
**18**	PT-A-7	PT	Male	55-59	ISCED-7	Nursing	Educator	Medical-surgical nursing
**19**	PT-A-8	PT	Male	30-34	ISCED-7	Nursing	Educator and researcher	Pediatrics, pediatric rehabilitation nursing
**20**	PT-A-9	PT	Female	60-64	ISCED-8	Nursing	Educator and researcher	Pediatrics, pediatric rehabilitation nursing
**21**	PT-A-10	PT	Male	30-34	ISCED-8	Medicine	Educator	Internist and intensivist, coordinator of simulation and emergency discipline
**22**	SI-A-1	SI	Female	30-34	ISCED-8	Nursing	Educator and researcher	Pediatric psychiatry nursing
**23**	SI-A-2	SI	Female	50-54	ISCED-7	Nursing	Educator and researcher	Geriatric nursing
**24**	SI-A-3	SI	Female	30-34	ISCED-7	Nursing	Educator and researcher	General nursing
**25**	SI-A-4	SI	Female	45-49	ISCED-7	Nursing	Educator and researcher	General nursing
**26**	SI-A-5	SI	Male	25-29	ISCED-7	Nursing	Educator and researcher	General nursing
**27**	SI-A-6	SI	Female	25-29	ISCED-7	Nursing	Educator and researcher	Informatics in nursing

Students

N	ID	Country	Gender	Age	Level of education	Study program	Current year
**1**	BE-S-1	BE	Female	18-24	ISCED-3	Dentistry	2
**2**	BE-S-2	BE	Male	18-24	ISCED-3	Pharmaceutical sciences	3
**3**	BE-S-3	BE	Male	18-24	ISCED-3	Pharmaceutical sciences	3
**4**	BE-S-4	BE	Female	18-24	ISCED-3	Biomedical sciences	3
**5**	NL-S-1	NL	Female	18-24	ISCED-6	Medicine	3
**6**	NL-S-2	NL	Female	18-24	ISCED-3	Medicine	2
**7**	NL-S-3	NL	Female	18-24	ISCED-3	Medicine	2
**8**	NL-S-4	NL	Female	18-24	ISCED-6	Health sciences	1
**9**	NL-S-5	NL	Female	18-24	ISCED-3	Medicine	3
**10**	NL-S-6	NL	Female	18-24	ISCED-6	Medicine	3
**11**	PT-S-1	PT	Male	18-24	ISCED-3	Nursing	2
**12**	PT-S-2	PT	Female	18-24	ISCED-3	Nursing	2
**13**	PT-S-3	PT	Female	18-24	ISCED-3	Physiotherapy	1
**14**	PT-S-4	PT	Female	18-24	ISCED-3	Medicine	2
**15**	PT-S-5	PT	Male	18-24	ISCED-3	Nursing	2
**16**	PT-S-6	PT	Female	18-24	ISCED-3	Nursing	4
**17**	PT-S-7	PT	Female	18-24	ISCED-3	Nursing	4
**18**	PT-S-8	PT	Male	18-24	ISCED-3	Nursing	4
**19**	PT-S-9	PT	Female	18-24	ISCED-3	Medicine	4

**20**	PT-S-10	PT	Female	18-24	ISCED-3	Medicine	5
**21**	SI-S-1	SI	Female	18-24	ISCED-3	Physiotherapy	3
**22**	SI-S-2	SI	Female	18-24	ISCED-3	Psychology	3
**23**	SI-S-3	SI	Female	18-24	ISCED-3	Pharmacy	3
**24**	SI-S-4	SI	Female	18-24	ISCED-3	Pharmacy	3
**25**	SI-S-5	SI	Male	18-24	ISCED-3	Nursing	3
**26**	SI-S-6	SI	Male	18-24	ISCED-3	Nursing	1
**27**	SI-S-7	SI	Female	18-24	ISCED-3	Nursing	3
